# Similar Connotation in Chronic Hepatitis B and Nonalcoholic Fatty Liver Patients with Dampness-Heat Syndrome

**DOI:** 10.1155/2013/793820

**Published:** 2013-04-08

**Authors:** Jianye Dai, Shujun Sun, Jianmei Cao, Yu Zhao, Huijuan Cao, Ningning Zheng, Junwei Fang, Yang Wang, Wei Zhang, Yongyu Zhang, Yiyang Hu, Zhiwei Cao

**Affiliations:** ^1^Center for Traditional Chinese Medicine and Systems Biology, Shanghai University of Traditional Chinese Medicine, Shanghai 201203, China; ^2^Institute of Liver Diseases, Shuguang Hospital, Key Laboratory of Liver and Kidney Diseases of Ministry of Education, Shanghai University of Traditional Chinese Medicine, Shanghai 201203, China; ^3^Liver Department, Longhua Hospital, Shanghai University of Traditional Chinese Medicine, Shanghai 200032, China; ^4^School of Life Sciences and Technology, Tongji University, Shanghai 200092, China

## Abstract

The phenomenon that the same syndrome turns up in different diseases appears in the sight of people around the world, which raises the thought for possibility of “Same Treatment for Different Diseases.” Actually, treatment based on ZHENG classification in Traditional Chinese Medicine could bring revelation for the former finding. The dampness-heat syndrome in chronic hepatitis B and nonalcoholic fatty liver is regarded as the breakthrough point. We discussed the molecular mechanism of similar connotation that exists in chronic hepatitis B and nonalcoholic fatty liver by metabonomics to give the modern understanding of dampness-heat syndrome. Both urine and serum metabolic profiling revealed that obvious differences existed between dampness-heat syndrome and non-dampness-heat syndrome but the commonality was proved to appear in chronic hepatitis B and nonalcoholic fatty liver patients with dampness-heat syndrome. Furthermore, disorder of body fluid metabolism, decline in digestive capacity, and imbalance of intestinal flora were found to be the new guiding for treatment, with the hope to provide the basis for Chinese personalized medicine.

## 1. Introduction

The phenomenon that different diseases reveal some homogeneity in symptoms [1, 2] is raising more and more attention, recently. More importantly, it suggests that different diseases with one or more similar pathological features may be treated with the same drugs [3, 4]. Actually, traditional Chinese medicine (TCM) participators have found and applied therapeutic principles of “Same Treatment for Different Diseases” for almost hundreds of years, which have been recorded in Treatise on Cold Damage Diseases (Shang Han Lun) and Synopsis of Prescriptions of the Golden Chamber (Jin Gui Yao Lue) [5, 6]. The same treatment for different diseases means applying the same treatment principles to patients with different kinds of disease only if with the same ZHENG [7]. What guides treatment is called ZHENG, a temporary state at one time and which is like a syndrome defined by symptoms and signs [8, 9]. The different diseases in western medicine can manifest in the same ZHENG and vice versa [10].

However, in TCM, one of the most common ZHENGs is dampness-heat syndrome (DH) which is thought to be caused by a combination of dampness and heat, either of external or of internal origin, with different manifestations according to location, for example, jaundice when dampness-heat accumulates in the liver and gallbladder and diarrhea for dampness-heat in the intestines [7]. Especially, it frequently occurs in Chronic hepatitis B (CHB) and nonalcoholic fatty liver (NFL), with 12.1% [11] and 37.1% [12] percentage respectively.

Since ZHENGs are frequently considered as phenotypes of a disease, researchers are mostly focused on the different ZHENGs in the same disease. However, we should pay more attention to the formerly mentioned phenomenon that the same ZHENG appears in different diseases. This part will bring more inspirations for broadening the application range of some drugs which are suitable for certain ZHENG. In this study, we discussed the molecular mechanisms of the DH in both CHB and NFL by metabonomics. It will be primarily serving for TCM diagnosis and treatment.

## 2. Experimental

### 2.1. Subjects and Experiment Design

Twenty healthy volunteers and eighty patients of dampness-heat syndrome chronic hepatitis B (DHHB), non-dampness-heat syndrome chronic hepatitis B (NDHHB), dampness-heat syndrome nonalcoholic fatty liver (DHFL), and non-dampness-heat syndrome nonalcoholic fatty liver (NDHFL) were enrolled in the study. The clinical study was approved by the local ethics committee and all of the recruited persons were given informed consent. Diagnostic standards of HB and FL patients were referred to “The guideline of prevention and treatment for chronic hepatitis B” [13] and “Guidelines for management of nonalcoholic fatty liver disease: an updated and revised edition” [14]. Cases meeting the diagnostic criteria for CHB and NFL, respectively, at 18–65 years of age and that would like to sign the informed consent form were included in the study. In addition, there is an exclusion criterion: (1) cases complicated with other hepatotropic virus hepatitis and alcoholic fatty liver, (2) chronic severe hepatitis, (3) HB and FL patients, associated with serious primary disease of heart, kidney, lung, endocrine, blood, or metabolic and gastrointestinal, or psychotic patients and (4) pregnant or lactating women. A junior medical physician made the initial diagnosis and recorded the information of four traditional examinations accurately and completely. Three more senior physicians (either chief or deputy physicians) subsequently confirmed the initial diagnosis by the records and gave the hierarchical results of typical degree. Only those cases that were identified as classical DH patients by both the junior and the senior physicians were included in the study to guarantee the correctness of ZHENG differentiation.

### 2.2. Chemicals and Drugs

N,O-Bis(trimethylsilyl)trifluoroacetamide (BSTFA with 1% TMCS), urease, L-2-chlorophenylalanine, and heptadecanoic acid were purchased from Sigma-Aldrich Co. LLC (USA). Methoxyamine hydrochloride, methanol, ethanol, myristic acid, chloroform, and pyridine were analytical grade from China National Pharmaceutical Group Corporation (Shanghai, China).

### 2.3. Sample Collection and Preparation

These patients were in ambulatory care. We collected the outpatients by the recommendation or reservation of doctor. A complete physical examination was given; the health condition was recorded on a scale including the information obtained through four traditional examination methods: looking, listening and smelling, asking, and touching at the patient's entry into the study.

And the urina sanguinis and serum were collected from all enrolled subjects. Urine and serum samples were stored at −80°C until GC-MS assay. All these samples were thawed in ice-water bath and vortex-mixed before analysis.

Each 1 mL aliquot of standard mixture or urine sample was placed into a screw tube, 10 min centrifugation (12,000 rpm) was given, and 150 *µ*L supernatants were transferred into another screw tube. After adding 70 *µ*L of urease (4 mg/mL) and vortex-mixing for 30 s, samples were conditioned at 37°C for 15 min to remove the urea. After adding 800 *µ*L of methanol and 10 *µ*L of myristic acid in methanol (1 mg/mL) and mixing for 1 min, the solution was centrifuged at 13,000 rpm for 10 min.

Each 100 *µ*L serum sample was transferred into a screw tube. After adding 10 *µ*L of L-2-chlorophenylalanine, 10 *µ*L of heptadecanoic acid, 300 *µ*L of solvent (methanol: chloroform, 3 : 1, V/V) and vortex-mixing for 30 s, samples were conditioned at −20°C for 10 min to precipitate protein. Then the solution was centrifuged at 13,000 rpm for 10 min.

Then a 200 *µ*L aliquot of supernatant was transferred into a GC vial and evaporated to dryness under N_2_ at 30°C. Methoxyamine in pyridine (15 mg/mL) was added to the GC vial vortex-mixed for 1 min, and the methoximation reaction was carried out for 90 min rocking in a shaker at 30°C; then 50 *µ*L of BSTFA plus 1% TMCS were added to the samples for trimethylsilylation for another 1 h at 70°C. At last, the solution was analyzed utilizing GC-MS after vortex for 30 s.

### 2.4. Data Acquisition

All GC-MS analyses were performed by a mass spectrometer 5975B (Agilent Technologies, USA) coupled to an Agilent 6890 (Agilent Technologies, USA) gas chromatography instrument. In the gas chromatographic system, a capillary column (Agilent J&W DB-5 ms Ultra Inert 30 m × 0.25 mm, film thickness 0.25 *μ*m) was used. Helium carrier gas was used at a constant flow rate of 1.0 mL × min^−1^. One *µ*L of derivatized samples was injected into the GC/MS instrument, and splitless injection mode was used. A programmed column temperature was optimized to acquire a well separation, which was demonstrated in Table 1. The temperatures of the injection port, the interface, and source temperature were set at 280°C, 260°C, and 230°C, respectively. The measurements were made with electron impact ionization (70 eV) in the full scan mode (m/z 30–550). The solvent delay time was set to 5 min. The GC-MS operating condition was the same as the previous experiment [15] except for the column temperature program.

### 2.5. Data Analysis

Due to experimental variations and column aging, shifts in retention time between fingerprints occurred. When the total ion current chromatograms (TICs) were obtained, peak-alignment or warping techniques were commonly applied to compensate for minor shifts in retention times. Thus, in the subsequently data processing, the same variable manifested synchronous information in every profile. So all the GC-MS raw files after being converted to CDF format via the software, which came with Agilent MSD workstation, were subsequently processed by the XCMS toolbox (http://metlin.scripps.edu/download/) using XCMS's default settings with the following exceptions: xcmsSet (full width at half-maximum: fwhm = 5; S/N cutoff value: snthresh = 10, max = 25), group (bw = 5). The resulting table (CSV file) was exported into Microsoft Excel (Microsoft Inc., USA), where normalization was performed prior to multivariate analyses. The resulting three-dimensional matrix involving peak index (RT-m/z pair), sample names (observations), and normalized peak area percent was introduced into Simca-P 11.5 Software (Umetrics, Umea, Sweden) for the analysis of principal component analysis (PCA), partial least squares discriminant analysis (PLS-DA), and orthogonal partial least squares (OPLS). Differential variables with VIP values [16] exceeding 1.5 between two different groups could be generated from OPLS loadings plot. Subsequently, those variables were further analyzed by Mann-Whitney *U* test to confirm the changed metabolites by SPSS 17.0 (SPSS, Chicago, IL, USA) with the threshold of *P* value set at 0.1. Those variables, then, were identified by searching in NIST 2005 database and verified by standards. The Kyoto Encyclopedia of Genes and Genomes (KEGG) (http://www.genome.ad.jp/kegg/) and Human Metabolome Database (HMDB) (http://www.hmdb.ca/) were used to give the biochemical interpretation of changed metabolites. Then, further analysis was performed by Metabolites Biological Role (MBRole) (http://csbg.cnb.csic.es/mbrole/).

## 3. Results

### 3.1. Clinical Information

The clinical information of all five groups was showed in Table 2, such as subjects' features and liver function indicators. There was no significant difference between every two syndromes in each disease.

### 3.2. Difference Analysis of Urinary Metabolic Profiles

**Figure 1:**
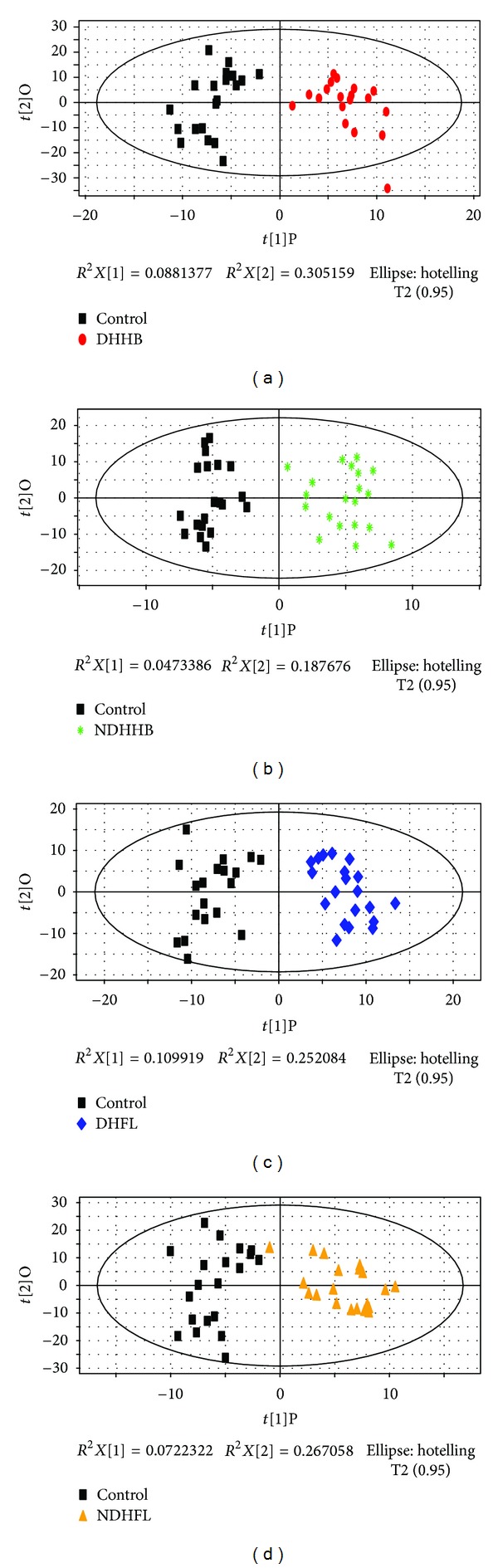
OPLS score plot of four ZHENGs compared to healthy control group by urinary metabolic profiles. (a) OPLS score plot between control and DHHB. (b) OPLS score plot between control and NDHHB. (c) OPLS score plot between control and DHFL. (d) OPLS score plot between control and NDHFL.

Figure 1 depicted score plot of orthogonal partial least squares (OPLS) of urine. It could be found that DHHB, NDHHB, DHFL, and NDHFL groups were clearly separated from the control group. The most meaningful characteristics were screened by orthogonal partial least squares (OPLS) loading plot analysis, which could effectively extract variables responsible for the separation by removing variables unrelated to pathological status. The quality of the model was characterized by two performance statistics, *R* ^2^ *Y* (cum) and *Q* ^2^ *Y* (cum), indicating the total explanation and predictability of the model [17]. The information of models was summarized in Table 7. Due to space limitation, the detailed information of selected urinary differential metabolites was summarized in Supplementary Information (SI) available at http://dx.doi.org/10.1155/2013/793820. But the brief information was listed in Table 3. Furthermore, the related pathways of each group were analyzed by KEGG and MBRole as followed, based on the selected differential metabolites (Table 4).

### 3.3. Difference Analysis of Serum Metabolic Profiles

**Figure 2:**
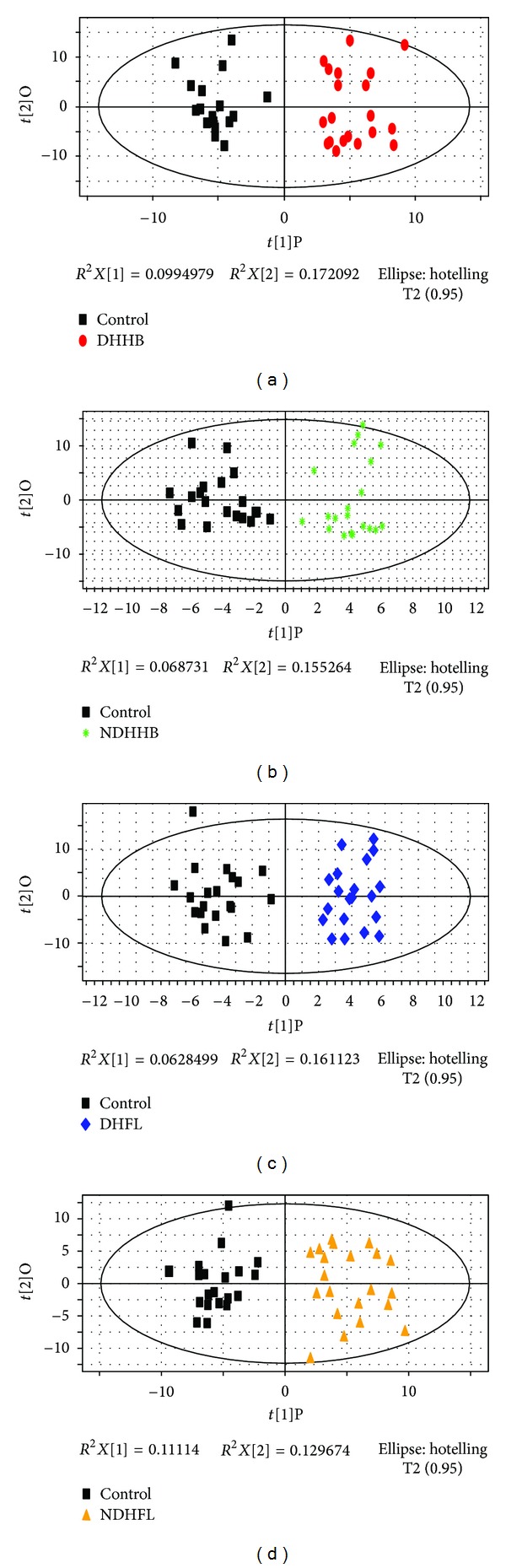
OPLS score plot of four ZHENGs compared to healthy control group by serum metabolic profiles. (a) OPLS score plot between control and DHHB. (b) OPLS score plot between control and NDHHB. (c) OPLS score plot between control and DHFL. (d) OPLS score plot between control and NDHFL.

The score plot of OPLS of serum was depicted in Figure 2 where it could be found that DHHB, NDHHB, DHFL, and NDHFL groups were clearly separated from the control group. Then the most meaningful characteristics of every group were screened out by OPLS loading analysis. The information of models was summarized in Table 7. The detailed information of serum differential metabolites was also summarized in SI, and the brief information was listed in Table 5. Furthermore, the corresponding pathways of each group were analyzed by KEGG and MBRole as follows, based on the selected differential metabolites (Table 6). 

### 3.4. Exploration of the Similar Connotation in CHB and NFL

**Figure 3:**
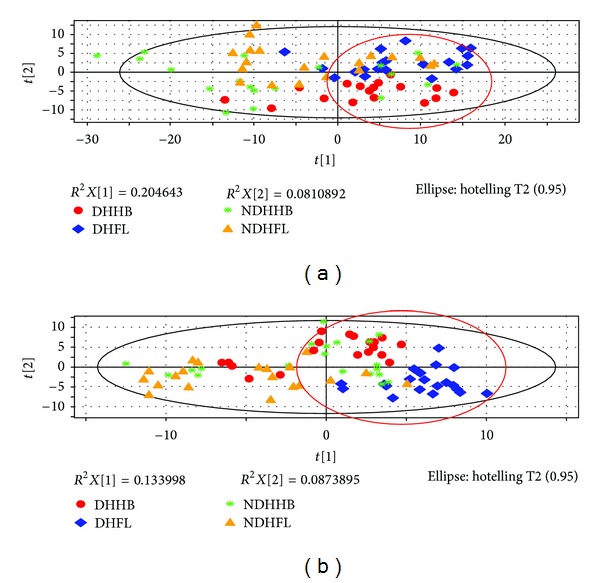
PLS-DA score plot of four ZHENGs. (a) PLS-DA score plot of urinary profiling. (b) PLS-DA score plot of serum profiling.

**Figure 4:**
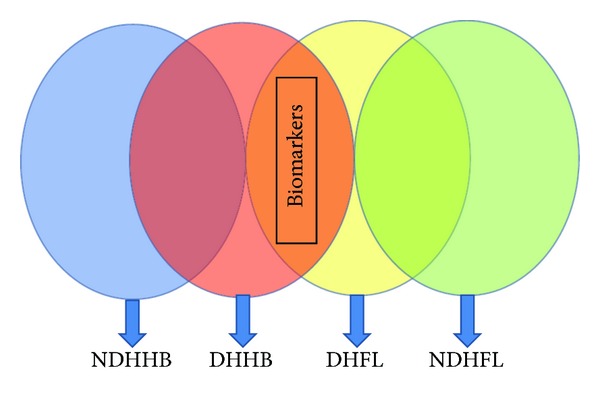
Schematic diagram of research approach for selection of DH.

The partial least squares discriminant analysis (PLS-DA) score plot of either urine or serum profiling could reveal that DHHB and DHFL clustered in a region (circled in Figure 3) and straggled with the other NDH groups. It might suggest that DHHB and DHFL have more commonalities which may be the connotation of DH. Given that all groups of selected markers may contain information of ZHENG and disease, the biomarkers of DH should be filtered further. Based on the thought of the “Same Disease with Different ZHENGs,” the potential biomarkers of a ZHENG in one disease could be selected by excluding the intersection of two or more ZHENGs of this disease. Therefore, DH potential biomarkers in CHB and NFL were selected, respectively, with the aforementioned process. Then we took advantage of the “Different Diseases with Same ZHENG,” to select the intersection of two upper groups of biomarkers as the final DH potential biomarkers (Table 8). The schematic diagram was shown by Figure 4. And the unique pathways of DH, which were obtained by the same method as above, were listed in Table 9. The respectively obtained urinary and serum information is integrated to interpret the connotation of DH exists in chronic hepatitis B and non-alcoholic fatty liver from the molecular level.

## 4. Discussion

The datasets from serum and urine were combined to get a global view of coanalysis. All the detected potential biomarkers, to a large extent, were caused by deregulated systemic changes and development or progression of DH. After analysis of the unique metabolites of DH, it was revealed that two most types of substances were carboxylic acid and alpha-amino acid. Eight biomarkers (acetic acid, succinic acid, and so on) were classified as carboxylic acid and four (glycine, L-lysine, L-asparagine, and aminolevulinic acid) as alpha-amino acid. Among these biomarkers of DH, only three metabolites (acetic acid, amino levulinic acid, and butyrate) were detected both in the serum and urine. Meanwhile, almost all biomarkers were uniquely serum-derived or urine-derived. This suggested the necessity to combine both serum and urine samples.

The characteristic metabolites of DH were hoped to be developed as the biomarkers. To achieve this goal, more cases are needed and another disease located in other organs should be incorporated to eliminate the impact of liver disease. After further validation, the filtered biomarkers could provide revelation to the standardization of DH classification and the subtype's distinction of CHB and NFL. But it is insufficient to explain the connotation of DH by only these characteristic metabolites. So we should expand our horizons to all the DH-related metabolites and pathways in our study.

**Figure 5:**
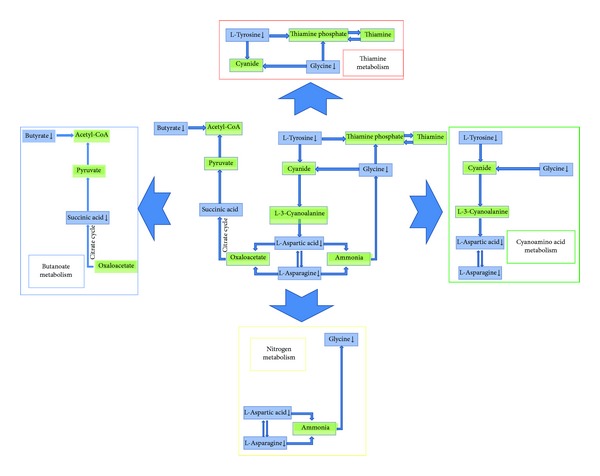
The schematic diagram of the unique pathways in DH.

**Figure 6:**
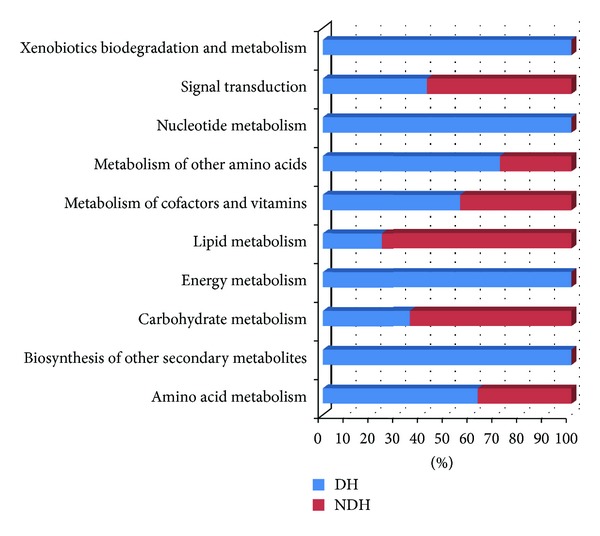
Comparison charts of metabolic functional modules between DH and NDH.

After coanalysis of serum and urinary information by KEGG and literature, the schematic diagram of the specific pathways in DH had been demonstrated in Figure 5. As shown, thiamine metabolism, nitrogen metabolism, cyanoamino acid metabolism, and butanoate metabolism were all downregulated. Furthermore, the statistics were performed for the metabolic modules of urinary and serum pathways in DH and NDH, as shown in Figure 6. We found that there was an obvious difference between DH and NDH in patients' metabolic levels. Especially, Biosynthesis of other secondary metabolites, energy metabolism, nucleotide metabolism, and xenobiotics biodegradation and metabolism were the unique metabolic changes. These may demonstrate the molecular mechanism of DH.

In our study, energy metabolism had been found, to have obvious changes, which was revealed by the down-regulated pathways of nitrogen metabolism and butanoate metabolism. As we all know is, energy metabolism ultimately linked to adenosine triphosphate (ATP). Downregulated Acetic acid and upregulated propanoic acid suggest that acetate kinase has been inhibited, which is closely related to ATP [18, 19]. Therefore the down-regulated pathway could be related to ATP insufficiency or decreased ATP enzyme activity, which could lead to a decrease in the Na^+^-K^+^-ATP enzyme activity. These all will result in sodium and potassium content disorders within cells and tissues. Furthermore, it causes liquid water imbalance, tissue edema, and other structural changes, to result in further energy supply shortage [20]. Some researchers have found a the decline of Na^+^-K^+^-ATP enzyme activity in DH patients' liver mitochondria and erythrocyte membrane [21, 22]. And Aquaporin-2, which is one of the water permeability transporter protein [23], has been reported to be reduced in DH [24]. Above all, body fluid metabolism disorder could be resulted by the accumulation of sodium elements in intracellular fluid, which sounds similar to the “water-dampness stagnation,” one of the incentives of the DH in TCM [25].

**Figure 7:**
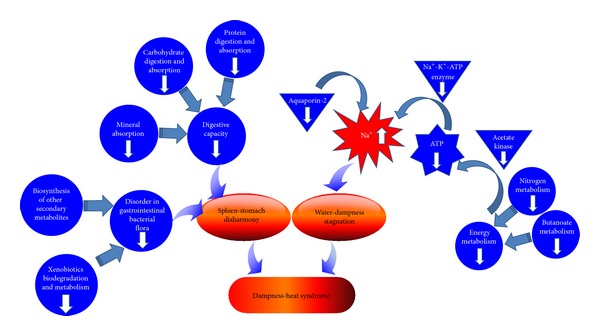
The schematic diagram of molecular mechanism of DH.

In addition, we found that carbohydrate digestion and absorption (D-glucose, acetate, butanoic acid), protein digestion and absorption (acetate, glycine, L-lysine, L-asparagine, butanoic acid), and mineral absorption (D-glucose, glycine, L-asparagine) were downregulated, which could be explained as decline in digestive capacity. In addition, the depressed microbial-related pathway, biosynthesis of other secondary metabolites [26], and xenobiotics biodegradation and metabolism [27, 28] would reveal the disorder in gastrointestinal bacterial flora, which may be the incentive of functional disturbance in digestion and absorption. In a word, the depressed digestive capacity and intestinal flora imbalance are both thought to be the main reason of “spleen-stomach disharmony,” another incentive of the DH [5, 6]. The schematic diagram of molecular mechanism of DH is demonstrated in Figure 7.

Edema and refractory are two common features of DH, which might be explained by the present results. Thiamine is one of eight members of the B family of vitamins, which is essential to the body to process carbohydrates. As an increase of dietary carbohydrate intake caused a decrease of plasma and urine levels of thiamine without affecting enzyme activities [29], the DH patients, who surfeit sweet and greasy food, will suffer more severe thiamine deficiency. This phenomenon has been proved by our results as down-regulated thiamine metabolism. However, thiamine deficiency would cause joint swelling [30, 31]. And the accumulation of sodium elements in intracellular fluid as former will make the edema more serious. To refractory, it is worth mentioning that down-regulated xenobiotics biodegradation and metabolism reveals the depression of scavenging endotoxin ability. A study showed that could be caused by inhibition bactericidal/permeability Increasing protein (BPI) mRNA expressing in moisture and heat environment [32], which is external factors of DH. Furthermore, in the vitality of the same bacteria or endotoxin activity conditions, the decrease in the synthesis and secretion of BPI could induce that the illness may develop or recovery is not easy to be cured.

Based on the former results, we could give our advice to develop new treatment for CHB and NFL. Firstly, the patients should be treated by considering the connotation of DH, when they appear the typical symptoms of DH such as red tongue and yellow greasy tongue coating. Secondly, the balance of sodium in intracellular fluid could be the new target for DH patients of CHB and NFL. Finally, regulating the function of spleen and stomach and balancing the intestinal flora would be the revelation for the treatment of CHB and NFL. Therefore, understanding the molecular mechanisms of DH in different diseases will be primarily serving for TCM diagnosis and treatment. This research firstly provides the evidence which needs to be proved by further research with more samples.

## 5. Conclusion

In this study, we conducted urine and serum metabonomics to explore the molecular mechanism of dampness-heat syndrome in CHB and NFL. After screening out the differential urine and serum metabolites of DHHB, NDHHB, DHFL, and NDHFL, the pathways of each group were analyzed. There were obvious differences found between DH and NDH in the metabolic level. And the commonality was proved to exist in CHB and NFL patients with DH. By two steps, the disease information was removed to reserve the characteristics of DH. Further studies showed that body fluid metabolism disorder resulted by the accumulation of sodium elements in intracellular fluid, depressed digestive capacity, and intestinal flora imbalance could be the main reasons for DH. We hope that the results could provide new revelation for treatment of CHB and NFL and a basis for expanding application of old drugs.

## Supplementary Material

The Supplementary material provides the detailed information of potential urinary and serum biomarkers, and the detailed pathway information based on integrated metabolites of Dampness-Heat Syndrome.

## Figures and Tables

**Table 1:** Temperature program of column incubator in GC/MS.

Project	Rate (°C/min)	Temperature (°C)	Hold time (min)
		70	2
Urine	2.5	160	0
	5	240	16

		80	2
Serum	5	240	0
	25	290	10

**Table 2:** The list of subjects' features and liver function indicators.

Indexes	Control	DHHB	NDHHB	DHFL	NDHFL
Gender (female/male)	3/17	4/16	3/17	2/18	6/14
Age (year)	39.20 ± 13.64	42.35 ± 15.23	43.05 ± 11.65	38.30 ± 17.24	42.15 ± 12.26
Stature (cm)	169.10 ± 7.77	170.10 ± 5.60	167.45 ± 5.39	171.9 ± 5.67	166.00 ± 8.55
Weight (kg)	61.15 ± 7.29	64.35 ± 10.68	66.40 ± 13.69	68.55 ± 7.93	61.70 ± 11.25
Alanine transaminase (U/L)	19.05 ± 5.25	69.30 ± 85.96	66.30 ± 65.57	43.70 ± 25.39	48.55 ± 32.56
Aspartate aminotransferase (U/L)	19.47 ± 4.01	52.50 ± 40.32	51.25 ± 44.92	29.40 ± 12.14	39.50 ± 24.81
Gamma glutamyltransferase (U/L)	18.53 ± 4.48	40.70 ± 26.04	85.75 ± 142.10	57.05 ± 50.66	32.20 ± 20.51
Alkaline phosphatase (U/L)	61.53 ± 15.47	96.20 ± 37.40	84.70 ± 24.97	66.30 ± 15.90	79.00 ± 17.32

**Table 3:** The compound number list of differential urinary metabolites in four ZHENGs.

Group	Compound number
U-DHHB	20
U-NDHHB	8
U-DHFL	23
U-NDHFL	12

**Table 4:** The information of changed pathways in urine of four ZHENGs.

Label	Group	Class^a^	*P* value^b^
Butanoate metabolism	U-DHHB	Carbohydrate metabolism	0.01
Starch and sucrose metabolism	U-DHHB	Carbohydrate metabolism	0.01
Pentose and glucuronate interconversions	U-DHHB	Carbohydrate metabolism	0.01
ABC transporters	U-DHHB	Signal transduction	0.00
Pentose and glucuronate interconversions	U-NDHHB	Carbohydrate metabolism	0.00
ABC transporters	U-NDHHB	Signal transduction	0.01
Phenylalanine metabolism	U-DHFL	Amino acid metabolism	0.03
Butanoate metabolism	U-DHFL	Carbohydrate metabolism	0.02
Galactose metabolism	U-DHFL	Carbohydrate metabolism	0.02
ABC transporters	U-DHFL	Signal transduction	0.09
Benzoate degradation	U-DHFL	Xenobiotics biodegradation and metabolism	0.04
Amino sugar and nucleotide sugar metabolism	U-NDHFL	Carbohydrate metabolism	0.01
Pentose and glucuronate interconversions	U-NDHFL	Carbohydrate metabolism	0.00

^a^The classes are categorized by KEGG.

^b^ *P* value is obtained from analysis of MBRole.

**Table 5:** The compound number list of differential serum metabolites in four ZHENGs.

Group	Compound number
S-DHHB	33
S-NDHHB	20
S-DHFL	26
S-NDHFL	23

**Table 6:** The information of changed pathways in serum of four ZHNEGs.

Label	Group	Class^a^	*P* value^b^
Alanine, aspartate, and glutamate metabolism	S-DHHB	Amino acid metabolism	0.02
Arginine and proline metabolism	S-DHHB	Amino acid metabolism	0.00
Glycine, serine, and threonine metabolism	S-DHHB	Amino acid metabolism	0.01
Lysine biosynthesis	S-DHHB	Amino acid metabolism	0.03
Lysine degradation	S-DHHB	Amino acid metabolism	0.00
Phenylalanine, tyrosine, and tryptophan biosynthesis	S-DHHB	Amino acid metabolism	0.02
Novobiocin biosynthesis	S-DHHB	Biosynthesis of other secondary metabolites	0.03
Streptomycin biosynthesis	S-DHHB	Biosynthesis of other secondary metabolites	0.02
Galactose metabolism	S-DHHB	Carbohydrate metabolism	0.00
Nitrogen metabolism	S-DHHB	Energy metabolism	0.00
Biosynthesis of unsaturated fatty acids	S-DHHB	Lipid metabolism	0.00
Fatty acid biosynthesis	S-DHHB	Lipid metabolism	0.06
Thiamine metabolism	S-DHHB	Metabolism of cofactors and vitamins	0.02
Cyanoamino acid metabolism	S-DHHB	Metabolism of other amino acids	0.00
Glutathione metabolism	S-DHHB	Metabolism of other amino acids	0.00
Pyrimidine metabolism	S-DHHB	Nucleotide metabolism	0.08
ABC transporters	S-DHHB	Signal transduction	0.00
Aminoacyl-tRNA biosynthesis	S-DHHB	Signal transduction	0.00
Two-component system	S-DHHB	Signal transduction	0.02
ABC transporters	S-DHHB	Signal transduction	0.01
Arginine and proline metabolism	S-NDHHB	Amino acid metabolism	0.00
Glycine, serine, and threonine metabolism	S-NDHHB	Amino acid metabolism	0.03
Lysine degradation	S-NDHHB	Amino acid metabolism	0.03
Galactose metabolism	S-NDHHB	Carbohydrate metabolism	0.02
Pentose and glucuronate interconversions	S-NDHHB	Carbohydrate metabolism	0.00
Biosynthesis of unsaturated fatty acids	S-NDHHB	Lipid metabolism	0.00
Fatty acid biosynthesis	S-NDHHB	Lipid metabolism	0.03
Nicotinate and nicotinamide metabolism	S-NDHHB	Metabolism of cofactors and vitamins	0.03
Glutathione metabolism	S-NDHHB	Metabolism of other amino acids	0.00
ABC transporters	S-NDHHB	Signal transduction	0.00
Aminoacyl-tRNA biosynthesis	S-NDHHB	Signal transduction	0.01
Arginine and proline metabolism	S-DHFL	Amino acid metabolism	0.06
Lysine degradation	S-DHFL	Amino acid metabolism	0.02
Glycolysis/Gluconeogenesis	S-DHFL	Carbohydrate metabolism	0.01
Nitrogen metabolism	S-DHFL	Energy metabolism	0.01
Thiamine metabolism	S-DHFL	Metabolism of cofactors and vitamins	0.01
Cyanoamino acid metabolism	S-DHFL	Metabolism of other amino acids	0.00
Glutathione metabolism	S-DHFL	Metabolism of other amino acids	0.01
Purine metabolism	S-DHFL	Nucleotide metabolism	0.01
ABC transporters	S-DHFL	Signal transduction	0.00
Aminoacyl-tRNA biosynthesis	S-DHFL	Signal transduction	0.00
Biosynthesis of unsaturated fatty acids	S-NDHFL	Lipid metabolism	0.00
Fatty acid biosynthesis	S-NDHFL	Lipid metabolism	0.00
ABC transporters	S-NDHFL	Signal transduction	0.01
Aminoacyl-tRNA biosynthesis	S-NDHFL	Signal transduction	0.01

^a^The classes are categorized by KEGG.

^b^ *P* value is obtained from analysis of MBRole.

**Table 7:** Summary of the modeling quality of OPLS analysis.

Name	Group	No^a^	*R* ^2^ *X*_cum_^b^	*R* ^2^ *Y*_cum_^c^	*Q* ^2^ *Y*_cum_^d^
1A	U-DHHB	1P + 2O^e^	0.50	0.89	0.70
1B	U-NDHHB	1P + 3O	0.56	0.90	0.48
1C	U-DHFL	1P + 3O	0.49	0.91	0.57
1D	U-NDHFL	1P + 2O	0.42	0.85	0.51
2A	S-DHHB	1P + 2O	0.36	0.91	0.67
2B	S-UDHHB	1P + 2O	0.31	0.88	0.58
2C	S-DHFL	1P + 2O	0.31	0.91	0.52
2D	S-NDHFL	1P + 2O	0.33	0.87	0.41

^a^No represents the number of components.

^b,c^ *R* ^2^ *X*_cum_ and *R* ^2^ *Y*_cum_ represent the cumulative sum of squares (SS) of all the *X*'s and *Y*'s explained by all extracted components.

^d^ *Q* ^2^ *Y*_cum_ is an estimate of how well the model predicts the *Y*'s.

^e^1P + 2O: one predictive component and two orthogonal components for establishing the OPLS model.

**Table 8:** The list of unique metabolites of DH.

Compound name	VIP^a^	*P* ^b^	FN^c^
*beta*-Aminoisobutyric acid	1.47	0.02	−1.52
*beta*-D-Glucopyranose	1.81	0.00	−1.79
1-Naphthol	1.79	0.01	−1.64
Butanedioic acid	2.00	0.00	−1.76
D-Glucose	1.17	0.02	+1.24
Glycine	1.86	0.00	−1.87
L-Asparagine	1.62	0.01	−1.60
L-Lysine	1.68	0.02	−1.68
Urea	1.84	0.01	+1.80
1H-Indole-3-acetic acid	1.65	0.00	−1.91
1H-Indole-3-butanoic acid	1.65	0.01	−1.59
(R)-Mandelic acid	1.72	0.01	−1.67
Acetic acid	1.70	0.00	−1.89
Butyrate	1.71	0.00	−1.69
Creatinine	1.71	0.01	−1.66
1-Cyclohexenecarboxylic acid	1.90	0.00	−1.74
Aminolevulinic acid	1.61	0.00	−1.79
Glutaconic acid	1.73	0.01	−1.63
Pteridine	1.66	0.00	−1.72
Pyrazine	1.58	0.00	−2.10
Succinic acid	1.88	0.01	−1.71

^a^VIP: variable importance in the project.

^b^ *P* value was obtained from Mann-Whitney test (ZHENGs compared to healthy control).

^c^FN is fold change of mean ranks calculated by the Mann-Whitney test (ZHENGs compared to healthy control). “+” means upregulated, “−” means downregulated.

**Table 9:** The list of unique pathways of DH in CHB and NFL.

Label	Sample	*P* value	Compound			
Cyanoamino acid metabolism	Serum	0.00	L-Tyrosine↓	Glycine↓	L-Asparagine↓	L-Aspartic acid↓
Nitrogen metabolism	Serum	0.01		Glycine↓	L-Asparagine↓	L-Aspartic acid↓
Thiamine metabolism	Serum	0.02	L-Tyrosine↓	Glycine↓		
Butanoate metabolism	Urine	0.01		Butyrate↓	Succinic acid↓	

The levels of differential metabolites were labeled with (↓) downregulated and (↑) upregulated.
