# Supplementary material for: Similar Connotation in Chronic Hepatitis B and Nonalcoholic Fatty Liver Patients with Dampness-Heat Syndrome
Source: Evid Based Complement Alternat Med. 2013 Apr 8;2013:793820. doi: 10.1155/2013/793820 (PMC3638589; doi:10.1155/2013/793820)
Supplement: Supplementary file 1 — The Supplementary material provides the detailed information of potential urinary and serum biomarkers, and the detailed pathway information based on integrated metabolites of Dampness-Heat Syndrome. [file 793820.f1.docx]

Supplementary Information

**Table 1. The table of detailed information of potential urinary biomarkers**

| Compound_name | Kegg^a^ | Group | VIP^b^ | P^c^ | FN^d^ |
| --- | --- | --- | --- | --- | --- |
| (R)-Mandelic acid | [C01983](http://www.genome.jp/dbget-bin/www_bget?cpd:C01983) | DHFL | 1.76 | 0.00 | -1.75 |
| 1-Cyclohexenecarboxylic acid | [C12101](http://www.genome.jp/dbget-bin/www_bget?cpd:C12101) | DHFL | 1.86 | 0.00 | -1.81 |
| 1H-Indole-3-acetic acid | [C00954](http://www.genome.jp/dbget-bin/www_bget?cpd:C00954) | DHFL | 1.72 | 0.00 | -2.09 |
| 1H-Indole-3-butanoic acid | [C11284](http://www.genome.jp/dbget-bin/www_bget?cpd:C11284) | DHFL | 1.68 | 0.00 | -1.68 |
| Acetic acid | C00033 | DHFL | 1.52 | 0.00 | -1.70 |
| Amino levulinic acid | [C00430](http://www.genome.jp/dbget-bin/www_bget?cpd:C00430) | DHFL | 1.54 | 0.00 | -1.82 |
| Benzoic acid | [C00180](http://www.genome.jp/dbget-bin/www_bget?cpd:C00180) | DHFL | 1.51 | 0.01 | -1.59 |
| Butyrate | [C00246](http://www.genome.jp/dbget-bin/www_bget?cpd:C00246) | DHFL | 1.54 | 0.01 | -1.65 |
| Creatinine | [C00791](http://www.genome.jp/dbget-bin/www_bget?cpd:C00791) | DHFL | 1.65 | 0.00 | -1.69 |
| D-Fructose | [C00095](http://www.genome.jp/dbget-bin/www_bget?cpd:C00095) | DHFL | 1.58 | 0.00 | -1.75 |
| d-Galactose | [C00124](http://www.genome.jp/dbget-bin/www_bget?cpd:C00124) | DHFL | 1.69 | 0.00 | -1.99 |
| D-Gluconic acid | [C00257](http://www.genome.jp/dbget-bin/www_bget?cpd:C00257) | DHFL | 1.51 | 0.00 | -1.70 |
| ethylene | [C06547](http://www.genome.jp/dbget-bin/www_bget?cpd:C06547) | DHFL | 1.51 | 0.01 | -1.57 |
| Glucaric acid | [C00818](http://www.genome.jp/dbget-bin/www_bget?cpd:C00818) | DHFL | 1.63 | 0.00 | -2.02 |
| Glutaconic acid | [C02214](http://www.genome.jp/dbget-bin/www_bget?cpd:C02214) | DHFL | 1.77 | 0.00 | -1.71 |
| Glutarate | [C00489](http://www.genome.jp/dbget-bin/www_bget?cpd:C00489) | DHFL | 1.57 | 0.01 | -1.61 |
| Gulonic acid | [C00800](http://www.genome.jp/dbget-bin/www_bget?cpd:C00800) | DHFL | 1.65 | 0.00 | -1.72 |
| Pseudo uridine | [C02067](http://www.genome.jp/dbget-bin/www_bget?cpd:C02067) | DHFL | 1.79 | 0.00 | -2.10 |
| Pteridine | [C07581](http://www.genome.jp/dbget-bin/www_bget?cpd:C07581) | DHFL | 1.57 | 0.00 | -1.73 |
| Pyrazinoic acid | [C19915](http://www.genome.jp/dbget-bin/www_bget?cpd:C19915) | DHFL | 1.53 | 0.00 | -2.19 |
| Succinic acid | [C00042](http://www.genome.jp/dbget-bin/www_bget?cpd:C00042) | DHFL | 1.90 | 0.00 | -1.81 |
| Tetradecanoic acid | [C06424](http://www.genome.jp/dbget-bin/www_bget?cpd:C06424) | DHFL | 1.68 | 0.00 | -1.74 |
| (R)-Mandelic acid | [C01983](http://www.genome.jp/dbget-bin/www_bget?cpd:C01983) | DHHB | 1.68 | 0.01 | -1.59 |
| 1-Cyclohexenecarboxylic acid | [C12101](http://www.genome.jp/dbget-bin/www_bget?cpd:C12101) | DHHB | 1.93 | 0.01 | -1.66 |
| 1H-Indole-3-acetic acid | [C00954](http://www.genome.jp/dbget-bin/www_bget?cpd:C00954) | DHHB | 1.58 | 0.00 | -1.73 |
| 1H-Indole-3-butanoic acid | [C11284](http://www.genome.jp/dbget-bin/www_bget?cpd:C11284) | DHHB | 1.61 | 0.02 | -1.50 |
| 2-Butenoic acid | [C01771](http://www.genome.jp/dbget-bin/www_bget?cpd:C01771) | DHHB | 1.81 | 0.02 | -1.53 |
| 3-Amino-1,2,4-triazole | [C11261](http://www.genome.jp/dbget-bin/www_bget?cpd:C11261) | DHHB | 1.74 | 0.02 | -1.51 |
| Acetic acid | [C00033](http://www.genome.jp/dbget-bin/www_bget?cpd:C00033) | DHHB | 1.87 | 0.00 | -2.07 |
| Amino levulinic acid | [C00430](http://www.genome.jp/dbget-bin/www_bget?cpd:C00430) | DHHB | 1.67 | 0.00 | -1.75 |
| benzene | [C01407](http://www.genome.jp/dbget-bin/www_bget?cpd:C01407) | DHHB | 1.88 | 0.01 | -1.56 |
| Benzophenone | [C06354](http://www.genome.jp/dbget-bin/www_bget?cpd:C06354) | DHHB | 1.58 | 0.02 | -1.53 |
| Butyrate | [C00246](http://www.genome.jp/dbget-bin/www_bget?cpd:C00246) | DHHB | 1.87 | 0.00 | -1.72 |
| Creatinine | C00791 | DHHB | 1.77 | 0.01 | -1.63 |
| d-Xylose | [C00181](http://www.genome.jp/dbget-bin/www_bget?cpd:C00181) | DHHB | 2.22 | 0.00 | -1.96 |
| Glutaconic acid | [C02214](http://www.genome.jp/dbget-bin/www_bget?cpd:C02214) | DHHB | 1.68 | 0.02 | -1.54 |
| Maltose | [C00208](http://www.genome.jp/dbget-bin/www_bget?cpd:C00208) | DHHB | 1.92 | 0.00 | +1.89 |
| Pteridine | [C07581](http://www.genome.jp/dbget-bin/www_bget?cpd:C07581) | DHHB | 1.75 | 0.00 | -1.71 |
| Pyrazinoic acid | [C19915](http://www.genome.jp/dbget-bin/www_bget?cpd:C19915) | DHHB | 1.62 | 0.00 | -2.00 |
| Ribitol | [C00474](http://www.genome.jp/dbget-bin/www_bget?cpd:C00474) | DHHB | 2.22 | 0.00 | -1.93 |
| Succinic acid | [C00042](http://www.genome.jp/dbget-bin/www_bget?cpd:C00042) | DHHB | 1.85 | 0.01 | -1.61 |
| Tartronic acid | [C02287](http://www.genome.jp/dbget-bin/www_bget?cpd:C02287) | DHHB | 2.28 | 0.00 | -2.10 |
| 2,3-Butanedione | [C00741](http://www.genome.jp/dbget-bin/www_bget?cpd:C00741) | NDHFL | 2.05 | 0.03 | +1.50 |
| 2-Butenoic acid | [C01771](http://www.genome.jp/dbget-bin/www_bget?cpd:C01595) | NDHFL | 1.87 | 0.01 | -1.31 |
| 3-Amino-1,2,4-triazole | C11261 | NDHFL | 1.87 | 0.06 | -1.41 |
| benzene | [C01407](http://www.genome.jp/dbget-bin/www_bget?cpd:C01407) | NDHFL | 1.91 | 0.01 | -1.32 |
| Chromium | [C06268](http://www.genome.jp/dbget-bin/www_bget?cpd:C06268) | NDHFL | 1.58 | 0.00 | -2.05 |
| d-Galactose | [C00124](http://www.genome.jp/dbget-bin/www_bget?cpd:C00124) | NDHFL | 1.64 | 0.01 | -1.31 |
| d-Xylose | C00181 | NDHFL | 2.03 | 0.02 | -1.28 |
| Ethanedioic acid | [C00209](http://www.genome.jp/dbget-bin/www_bget?cpd:C00209) | NDHFL | 1.79 | 0.04 | -1.44 |
| Propanedioic acid | [C00383](http://www.genome.jp/dbget-bin/www_bget?cpd:C00383) | NDHFL | 1.49 | 0.01 | +1.64 |
| Ribitol | C00474 | NDHFL | 2.04 | 0.02 | -1.27 |
| Ribonic acid | [C01685](http://www.genome.jp/dbget-bin/www_bget?cpd:C01685) | NDHFL | 1.69 | 0.02 | +1.29 |
| Tartronic acid | C02287 | NDHFL | 2.11 | 0.01 | -1.34 |
| 2,3-Butanedione | [C00741](http://www.genome.jp/dbget-bin/www_bget?cpd:C00741) | NDHHB | 1.89 | 0.02 | +1.56 |
| 3-Amino-1,2,4-triazole | C11261 | NDHHB | 2.31 | 0.03 | -1.48 |
| D-Gluconic acid | [C00257](http://www.genome.jp/dbget-bin/www_bget?cpd:C00257) | NDHHB | 1.87 | 0.00 | +1.85 |
| d-Xylose | C00181 | NDHHB | 2.15 | 0.01 | -1.57 |
| Glycine | [C00037](http://www.genome.jp/dbget-bin/www_bget?cpd:C00037) | NDHHB | 2.11 | 0.01 | +1.62 |
| Ribitol | C00474 | NDHHB | 2.19 | 0.01 | -1.57 |
| Tartronic acid | C02287 | NDHHB | 2.21 | 0.00 | -1.66 |
| Vanillylmandelic acid | [C05584](http://www.genome.jp/dbget-bin/www_bget?cpd:C05584) | NDHHB | 1.94 | 0.05 | +1.42 |

**a This is the Compound ID in KEGG.**

**b VIP, variable importance in the project.**

**c P value was obtained from Mann-Whitney test (ZHENGs compared to Healthy Control).**

**d FN is fold change of mean ranks calculated by the Mann-Whitney test (ZHENGs compared to Healthy Control). “+” means up-regulated, “-” means down-regulated.**

**Table 2. The table of detailed information of potential serum biomarkers**

| Compound_name | Kegg^a^ | ZHENG | VIP^b^ | P^c^ | FN^d^ |
| --- | --- | --- | --- | --- | --- |
| (S)-N-Methylcoclaurine | [C05176](http://www.genome.jp/dbget-bin/www_bget?cpd:C05176) | DHFL | 1.49 | 0.02 | -1.27 |
| .beta.-Amino isobutyric acid | [C03665](http://www.genome.jp/dbget-bin/www_bget?cpd:C03665) | DHFL | 1.66 | 0.02 | -1.54 |
| .beta.-D-Glucopyranose | [C08473](http://www.genome.jp/dbget-bin/www_bget?cpd:C08473) | DHFL | 1.75 | 0.00 | -1.72 |
| 1-Naphthol | [C11714](http://www.genome.jp/dbget-bin/www_bget?cpd:C11714) | DHFL | 2.07 | 0.00 | -1.67 |
| 5,7-Dihydroxychromone | [C09001](http://www.genome.jp/dbget-bin/www_bget?cpd:C09001) | DHFL | 1.53 | 0.03 | +1.49 |
| Acetic acid | C00033 | DHFL | 1.65 | 0.02 | -1.54 |
| Aminomalonic acid | [C00872](http://www.genome.jp/dbget-bin/www_bget?cpd:C00872) | DHFL | 1.78 | 0.01 | -1.66 |
| Arabinofuranose | [C06115](http://www.genome.jp/dbget-bin/www_bget?cpd:C00791) | DHFL | 1.72 | 0.01 | -1.59 |
| Benzophenone | [C06354](http://www.genome.jp/dbget-bin/www_bget?cpd:C00791) | DHFL | 1.51 | 0.05 | -1.41 |
| Butanedioic acid | [C04067](http://www.genome.jp/dbget-bin/www_bget?cpd:C04067) | DHFL | 2.01 | 0.00 | -1.68 |
| Butyrate | C00246 | DHFL | 2.47 | 0.00 | +1.92 |
| d-Glucose | [C00031](http://www.genome.jp/dbget-bin/www_bget?cpd:C00031) | DHFL | 1.23 | 0.03 | +1.19 |
| Glycine | C00037 | DHFL | 1.94 | 0.00 | -1.74 |
| Hexadecanoic acid | C00249 | DHFL | 2.06 | 0.00 | +1.79 |
| Hexanoic acid | C01585 | DHFL | 2.00 | 0.00 | +1.72 |
| L-Asparagine | [C00152](http://www.genome.jp/dbget-bin/www_bget?cpd:C00152) | DHFL | 1.66 | 0.01 | -1.58 |
| L-Leucine | C00123 | DHFL | 1.59 | 0.02 | +1.52 |
| L-Lysine | [C00047](http://www.genome.jp/dbget-bin/www_bget?cpd:C00047) | DHFL | 1.49 | 0.03 | -1.46 |
| L-Ornithine | [C00077](http://www.genome.jp/dbget-bin/www_bget?cpd:C00077) | DHFL | 1.84 | 0.01 | -1.61 |
| L-Valine | [C00183](http://www.genome.jp/dbget-bin/www_bget?cpd:C00082) | DHFL | 1.60 | 0.02 | +1.54 |
| Phosphoramidate | [C02306](http://www.genome.jp/dbget-bin/www_bget?cpd:C02306) | DHFL | 1.44 | 0.09 | -1.34 |
| Propanoic acid | C00163 | DHFL | 1.89 | 0.00 | +1.77 |
| Urea | [C00086](http://www.genome.jp/dbget-bin/www_bget?cpd:C00086) | DHFL | 1.63 | 0.01 | +1.56 |
| Uric acid | [C00366](http://www.genome.jp/dbget-bin/www_bget?cpd:C00366) | DHFL | 2.66 | 0.00 | +2.13 |
| Valeric acid | [C00803](http://www.genome.jp/dbget-bin/www_bget?cpd:C00803) | DHFL | 1.56 | 0.02 | +1.51 |
| .beta.-Amino isobutyric acid | [C03665](http://www.genome.jp/dbget-bin/www_bget?cpd:C00181) | DHHB | 1.27 | 0.03 | -1.49 |
| .beta.-D-Glucopyranose | [C08473](http://www.genome.jp/dbget-bin/www_bget?cpd:C00181) | DHHB | 1.86 | 0.00 | -1.86 |
| 1,4-Butanediamine | [C00134](http://www.genome.jp/dbget-bin/www_bget?cpd:C00134) | DHHB | 1.06 | 0.05 | -1.42 |
| 11-Eicosenoic acid | [C16526](http://www.genome.jp/dbget-bin/www_bget?cpd:C00209) | DHHB | 2.00 | 0.00 | -1.94 |
| 1H-Indole | [C00463](http://www.genome.jp/dbget-bin/www_bget?cpd:C00209) | DHHB | 1.34 | 0.01 | -1.57 |
| 1H-Pyrrole | [C19907](http://www.genome.jp/dbget-bin/www_bget?cpd:C00209) | DHHB | 1.88 | 0.00 | -1.91 |
| 1-Naphthol | [C11714](http://www.genome.jp/dbget-bin/www_bget?cpd:C11714) | DHHB | 1.50 | 0.01 | -1.61 |
| 2-Piperidinecarboxylic acid | [C00408](http://www.genome.jp/dbget-bin/www_bget?cpd:C00408) | DHHB | 1.27 | 0.05 | -1.44 |
| 4-Hydroxybutanoic acid | [C00989](http://www.genome.jp/dbget-bin/www_bget?cpd:C00989) | DHHB | 1.71 | 0.01 | -1.56 |
| 9,12-Octadecadienoic acid | [C01595](http://www.genome.jp/dbget-bin/www_bget?cpd:C01595) | DHHB | 1.47 | 0.01 | -1.66 |
| Acetylene | [C01548](http://www.genome.jp/dbget-bin/www_bget?cpd:C01548) | DHHB | 1.83 | 0.00 | +1.89 |
| Amino levulinic acid | [C00430](http://www.genome.jp/dbget-bin/www_bget?cpd:C00430) | DHHB | 1.13 | 0.04 | -1.45 |
| Butanedioic acid | [C04067](http://www.genome.jp/dbget-bin/www_bget?cpd:C00037) | DHHB | 1.99 | 0.00 | -1.83 |
| Cadaverine | [C01672](http://www.genome.jp/dbget-bin/www_bget?cpd:C00037) | DHHB | 1.70 | 0.00 | -2.40 |
| D-Glucitol | [C00794](http://www.genome.jp/dbget-bin/www_bget?cpd:C00794) | DHHB | 1.41 | 0.02 | -1.54 |
| d-Glucose | [C00031](http://www.genome.jp/dbget-bin/www_bget?cpd:C00031) | DHHB | 1.11 | 0.02 | +1.28 |
| Epimelibiose | [C05400](http://www.genome.jp/dbget-bin/www_bget?cpd:C00249) | DHHB | 2.32 | 0.00 | -2.29 |
| Ethanimidic acid | [C00209](http://www.genome.jp/dbget-bin/www_bget?cpd:C00249) | DHHB | 1.72 | 0.00 | +2.19 |
| Glutamine | [C00303](http://www.genome.jp/dbget-bin/www_bget?cpd:C00303) | DHHB | 1.48 | 0.01 | -1.63 |
| Glycine | [C00037](http://www.genome.jp/dbget-bin/www_bget?cpd:C01585) | DHHB | 1.78 | 0.00 | -2.00 |
| Hexanoic acid | [C01585](http://www.genome.jp/dbget-bin/www_bget?cpd:C01585) | DHHB | 1.16 | 0.05 | +1.41 |
| L-Asparagine | [C00152](http://www.genome.jp/dbget-bin/www_bget?cpd:C00152) | DHHB | 1.58 | 0.01 | -1.62 |
| L-Aspartic acid | [C00049](http://www.genome.jp/dbget-bin/www_bget?cpd:C00049) | DHHB | 1.73 | 0.00 | -1.82 |
| L-Lysine | [C00047](http://www.genome.jp/dbget-bin/www_bget?cpd:C00047) | DHHB | 1.87 | 0.00 | -1.89 |
| L-Proline | [C00148](http://www.genome.jp/dbget-bin/www_bget?cpd:C00148) | DHHB | 2.29 | 0.00 | -2.33 |
| L-Tyrosine | [C00082](http://www.genome.jp/dbget-bin/www_bget?cpd:C00082) | DHHB | 1.77 | 0.00 | -1.88 |
| Mercaptoacetic acid | [C02086](http://www.genome.jp/dbget-bin/www_bget?cpd:C02086) | DHHB | 1.20 | 0.03 | +1.50 |
| Myo-Inositol | [C00137](http://www.genome.jp/dbget-bin/www_bget?cpd:C00137) | DHHB | 1.37 | 0.01 | -1.57 |
| Octadecanoic acid | [C01530](http://www.genome.jp/dbget-bin/www_bget?cpd:C01530) | DHHB | 1.87 | 0.00 | -1.97 |
| Oleic acid | [C00712](http://www.genome.jp/dbget-bin/www_bget?cpd:C00712) | DHHB | 1.90 | 0.00 | -1.89 |
| Pyridine | [C00747](http://www.genome.jp/dbget-bin/www_bget?cpd:C00747) | DHHB | 1.07 | 0.02 | +1.53 |
| Thioredoxin disulfide | [C00343](http://www.genome.jp/dbget-bin/www_bget?cpd:C00343) | DHHB | 1.52 | 0.00 | -1.72 |
| Urea | [C00086](http://www.genome.jp/dbget-bin/www_bget?cpd:C00086) | DHHB | 2.04 | 0.00 | +2.04 |
| 11-Eicosenoic acid | [C16526](http://www.genome.jp/dbget-bin/www_bget?cpd:C16526) | NDHFL | 1.70 | 0.01 | -1.57 |
| 1H-Pyrrole | [C19907](http://www.genome.jp/dbget-bin/www_bget?cpd:C00148) | NDHFL | 1.76 | 0.00 | -1.71 |
| 4-Hydroxybutanoic acid | [C00989](http://www.genome.jp/dbget-bin/www_bget?cpd:C00148) | NDHFL | 1.60 | 0.03 | -1.46 |
| 9,12-Octadecadienoic acid | C01595 | NDHFL | 1.91 | 0.02 | +1.51 |
| Alanine | [C00041](http://www.genome.jp/dbget-bin/www_bget?cpd:C00041) | NDHFL | 1.67 | 0.02 | +1.52 |
| Benzamide | [C09815](http://www.genome.jp/dbget-bin/www_bget?cpd:C09815) | NDHFL | 1.65 | 0.03 | -1.48 |
| Benzophenone | C06354 | NDHFL | 1.63 | 0.02 | +1.52 |
| Butyrate | C00246 | NDHFL | 1.59 | 0.00 | +1.72 |
| Epimelibiose | C05400 | NDHFL | 1.59 | 0.01 | -1.57 |
| Ethanedioic acid | C00209 | NDHFL | 1.67 | 0.00 | +1.74 |
| Hexadecanoic acid | C00249 | NDHFL | 1.59 | 0.01 | +1.63 |
| Hexanoic acid | C01585 | NDHFL | 1.73 | 0.00 | +1.94 |
| L-Leucine | [C00123](http://www.genome.jp/dbget-bin/www_bget?cpd:C00712) | NDHFL | 1.96 | 0.00 | +1.89 |
| L-Proline | [C00148](http://www.genome.jp/dbget-bin/www_bget?cpd:C00712) | NDHFL | 1.87 | 0.00 | -1.71 |
| Octadecanoic acid | [C01530](http://www.genome.jp/dbget-bin/www_bget?cpd:C01530) | NDHFL | 1.71 | 0.01 | +1.57 |
| Oleic acid; | C00712 | NDHFL | 1.60 | 0.03 | -1.49 |
| Phosphoramidate | [C02306](http://www.genome.jp/dbget-bin/www_bget?cpd:C02306) | NDHFL | 1.51 | 0.02 | -1.55 |
| Propanoic acid | C00163 | NDHFL | 1.54 | 0.05 | +1.42 |
| Pyridine | [C00747](http://www.genome.jp/dbget-bin/www_bget?cpd:C00747) | NDHFL | 1.73 | 0.01 | +1.65 |
| Ribitol | [C00474](http://www.genome.jp/dbget-bin/www_bget?cpd:C00163) | NDHFL | 1.53 | 0.03 | -1.48 |
| Thioredoxin disulfide | [C00343](http://www.genome.jp/dbget-bin/www_bget?cpd:C00163) | NDHFL | 1.59 | 0.01 | -1.56 |
| Uric acid | [C00366](http://www.genome.jp/dbget-bin/www_bget?cpd:C00366) | NDHFL | 1.55 | 0.00 | +1.78 |
| Valeric acid | [C00803](http://www.genome.jp/dbget-bin/www_bget?cpd:C00803) | NDHFL | 1.93 | 0.00 | +1.88 |
| 1,4-Butanediamine | [C00134](http://www.genome.jp/dbget-bin/www_bget?cpd:C00134) | NDHHB | 1.56 | 0.02 | -1.52 |
| 1H-Pyrrole | C19907 | NDHHB | 1.82 | 0.01 | -1.67 |
| 2-Piperidinecarboxylic acid | [C00408](http://www.genome.jp/dbget-bin/www_bget?cpd:C00408) | NDHHB | 1.65 | 0.02 | -1.53 |
| 4,4'-Dihydroxydiphenylmethane | [C14298](http://www.genome.jp/dbget-bin/www_bget?cpd:C14298) | NDHHB | 1.66 | 0.01 | -1.57 |
| 9,12-Octadecadienoic acid | C01595 | NDHHB | 1.50 | 0.02 | +1.55 |
| Amino levulinic acid | [C00430](http://www.genome.jp/dbget-bin/www_bget?cpd:C00430) | NDHHB | 1.54 | 0.01 | -1.57 |
| Butyrate | [C00246](http://www.genome.jp/dbget-bin/www_bget?cpd:C00474) | NDHHB | 1.37 | 0.07 | +1.39 |
| Cadaverine | [C01672](http://www.genome.jp/dbget-bin/www_bget?cpd:C00474) | NDHHB | 1.71 | 0.02 | -1.52 |
| Creatinine | [C00791](http://www.genome.jp/dbget-bin/www_bget?cpd:C00474) | NDHHB | 2.04 | 0.00 | -2.21 |
| Epimelibiose | C05400 | NDHHB | 1.46 | 0.09 | +1.34 |
| Ethanedioic acid | C00209 | NDHHB | 1.43 | 0.08 | +1.37 |
| Hexadecanoic acid | [C00249](http://www.genome.jp/dbget-bin/www_bget?cpd:C00249) | NDHHB | 1.96 | 0.00 | +1.79 |
| Hexanoic acid | C01585 | NDHHB | 1.51 | 0.06 | +1.40 |
| L-Aspartic acid | [C00049](http://www.genome.jp/dbget-bin/www_bget?cpd:C00049) | NDHHB | 2.10 | 0.00 | -1.82 |
| L-Leucine | [C00123](http://www.genome.jp/dbget-bin/www_bget?cpd:C00123) | NDHHB | 1.39 | 0.09 | +1.33 |
| L-Ornithine | [C00077](http://www.genome.jp/dbget-bin/www_bget?cpd:C00077) | NDHHB | 1.86 | 0.00 | +1.72 |
| L-Proline | C00148 | NDHHB | 2.11 | 0.00 | -1.72 |
| Myo-Inositol | [C00137](http://www.genome.jp/dbget-bin/www_bget?cpd:C00137) | NDHHB | 2.09 | 0.00 | -1.69 |
| Oleic acid | C00712 | NDHHB | 1.60 | 0.03 | +1.47 |
| Propanoic acid | [C00163](http://www.genome.jp/dbget-bin/www_bget?cpd:C00163) | NDHHB | 1.68 | 0.02 | +1.55 |

**a This is the Compound ID in KEGG.**

**b VIP, variable importance in the project.**

**c P value was obtained from Mann-Whitney test (ZHENGs compared to Healthy Control).**

**d FN is fold change of mean ranks calculated by the Mann-Whitney test (ZHENGs compared to Healthy Control). “+” means up-regulated, “-” means down-regulated.**

**Table 3. The table of detailed information of pathway based on integrated metabolites of DH**

| Pathway | Compounds | | | | | | |
| --- | --- | --- | --- | --- | --- | --- | --- |
| Biosynthesis of  secondary metabolites (7) | D-Glucose | 5-Aminolevulinate | L-Asparagine | Acetate | Glycine | Succinate | L-Lysine |
| Microbial metabolism  in diverse environments (6) | Acetate | Glycine | Succinate | L-Lysine | Urea | (R)- Mandelate |  |
| Protein digestion  and absorption (5) | Acetate | Glycine | L-Asparagine | Butanoic  acid | L-Lysine |  |  |
| ABC transporters (5) | D-Glucose | 5-Aminolevulinate | Glycine | L-Lysine | Urea |  |  |
| Carbohydrate digestion  and absorption (3) | D-Glucose | Acetate | Butanoic acid |  |  |  |  |
| Aminoacyl-tRNA  biosynthesis (3) | Glycine | L-Lysine | L-Asparagine |  |  |  |  |
| Mineral absorption (3) | D-Glucose | Glycine | L-Asparagine |  |  |  |  |
| Arginine and proline  metabolism (2) | Urea | Creatinine |  |  |  |  |  |
| Two-component system (2) | D-Glucose | Succinate |  |  |  |  |  |
| Carbon fixation pathways  in prokaryotes (2) | Acetate | Succinate |  |  |  |  |  |
| Glycolysis /Gluconeogenesis (2) | D-Glucose | Acetate |  |  |  |  |  |
| Lysine degradation (2) | Glycine | L-Lysine |  |  |  |  |  |
| Biotin metabolism (2) | L-Lysine | Urea |  |  |  |  |  |
| Purine metabolism (2) | Glycine | Urea |  |  |  |  |  |
| Methane metabolism (2) | Acetate | Glycine |  |  |  |  |  |
| Porphyrin and chlorophyll metabolism (2) | Glycine | 5-Aminolevulinate |  |  |  |  |  |
| Glycine, serine and  threonine metabolism (2) | Glycine | 5-Aminolevulinate |  |  |  |  |  |
| Nitrogen metabolism (2) | Glycine | L-Asparagine |  |  |  |  |  |
| Glyoxylate and dicarboxylate metabolism (2) | Glycine | Succinate |  |  |  |  |  |
| Alanine, aspartate and glutamate metabolism (2) | Succinate | L-Asparagine |  |  |  |  |  |
| Cyanoamino acid  metabolism (2) | Glycine | L-Asparagine |  |  |  |  |  |
| Butanoate metabolism (2) | Succinate | Butanoic acid |  |  |  |  |  |
